# Kaposi sarcoma associated herpesvirus (KSHV) induces AKT hyperphosphorylation, bortezomib-resistance and GLUT-1 plasma membrane exposure in THP-1 monocytic cell line

**DOI:** 10.1186/1756-9966-32-79

**Published:** 2013-10-23

**Authors:** Roberta Gonnella, Roberta Santarelli, Antonella Farina, Marisa Granato, Gabriella D’Orazi, Alberto Faggioni, Mara Cirone

**Affiliations:** 1 Department of Experimental Medicine, Istituto Pasteur Fondazione Cenci Bolognetti, La Sapienza University, 00161 Rome, Italy; 2 Department of Medical, Oral and Biotechnological Sciences, University “G. d’Annunzio”, 66013 Chieti, Italy; 3 Department of Experimental Oncology, Regina Elena National Cancer Institute, 00158 Rome, Italy

**Keywords:** KSHV, PEL, THP-1, AKT, Bortezomib, GLUT1, 2-Deoxy-D-Glucose

## Abstract

**Background:**

Phosphatidylinositol-3-kinase (PI3K)/AKT/mammalian target of rapamycin (mTOR) signaling pathway regulates multiple cellular processes such as cell proliferation, evasion from apoptosis, migration, glucose metabolism, protein synthesis and proper differentiation in immune cells. Kaposi sarcoma-associated herpesvirus (KSHV), an oncogenic virus associated with several human malignancies, expresses a variety of latent and lytic proteins able to activate PI3K/AKT pathway, promoting the growth of infected cells and a successful viral infection.

**Results:**

We found that KSHV latent infection of THP-1 cells, a human monocytic cell line derived from an acute monocytic leukemia patient, resulted in an increase of AKT phoshorylation, not susceptible to bortezomib-induced dephosphorylation, compared to the mock-infected THP-1. Accordingly, THP-1-infected cells displayed increased resistance to the bortezomib cytotoxic effect in comparison to the uninfected cells, which was counteracted by pre-treatment with AKT-specific inhibitors. Finally, AKT hyperactivation by KSHV infection correlated with plasma membrane exposure of glucose transporter GLUT1, particularly evident during bortezomib treatment. GLUT1 membrane trafficking is a characteristic of malignant cells and underlies a change of glucose metabolism that ensures the survival to highly proliferating cells and render these cells highly dependent on glycolysis. GLUT1 membrane trafficking in KSHV-infected THP-1 cells indeed led to increased sensitivity to cell death induced by the glycolysis inhibitor 2-Deoxy-D-glucose (2DG), further potentiated by its combination with bortezomib.

**Conclusions:**

KSHV confers to the THP-1 infected cells an oncogenic potential by altering the phosphorylation, expression and localization of key molecules that control cell survival and metabolism such as AKT and GLUT1. Such modifications in one hand lead to resistance to cell death induced by some chemotherapeutic drugs such as bortezomib, but on the other hand, offer an Achilles heel, rendering the infected cells more sensitive to other treatments such as AKT or glycolysis inhibitors. These therapeutic strategies can be exploited in the anticancer therapy of KSHV-associated malignancies.

## Introduction

Kaposi sarcoma-associated herpesvirus (KSHV) is a human gammaherpesvirus found in all forms of Kaposi’s sarcoma (KS) and is also highly associated with two lymphoproliferative disorders that are primary effusion lymphoma (PEL) and multicentric Castleman’s disease (MCD) [1]. KSHV is able to infect a variety of non haematological and haematological cells such as B and T lymphocytes, monocytes, macrophages and dendritic cells (DC) that express the known KSHV receptors [2-6], such as proteoglycan heparan sulphates (HS), DC-SIGN and some integrins [7-10]. THP-1 is a monocytic cell line derived from an acute monocytic leukemia patient whose infection by KSHV has been previously reported [11,12]. These cells support a latent viral infection that implies the expression of few viral proteins in the majority of the infected cells that is sufficient to subvert the expression of monocyte activation markers and influence the cytokine release [12]. Among the molecular pathways altered in tumor cells harboring KSHV, or following KSHV de-novo infection is phosphatidylinositol-3-kinase (PI3K)/AKT/mammalian target of rapamycin (mTOR) [13,14], which is an ubiquitous pathway that controls cell survival and cell metabolism [15,16]. PI3Ks are divided into four classes that have different substrate specificities. Among them class I catalyzes the phosphorylation of phosphatidylinositol-4,5-bisphosphate (PIP2) into phosphatidylinositol-3,4,5-triphosphate (PIP3) which has a pivotal role in the control of cell growth, survival and motility. PIP3 dephosphorylation is catalyzed by phosphatase and tensin homolog (PTEN), which is a phosphatase frequently mutated or deleted in cancers [17]. The hyperactivation of AKT, due to activation of class I PI3K or to PTEN mutations/deletion, promotes cellular proliferation, glucose metabolism, protein synthesis and increases evasion from apoptosis induction by inactivating pro-apoptotic proteins [18,19]. AKT pathway can be activated in KSHV-infected cells as a consequence of the expression of viral proteins that interfere with PTEN [20,21], or directly activate PI3K [14]. AKT stimulates glycolysis by increasing the expression and membrane translocation of glucose transporters (i.e., GLUT1) which correlates with decreased response to therapy, as also reported by our studies [22], and overall survival in many cancer patients [16]. GLUT1 up-regulation and membrane exposure is indeed intricately linked to cancer progression since cancer cells need to support high proliferation rates and thus require efficient biosynthesis of macromolecules [23]. Consequently, signals leading to increased proliferation must also drive the necessary adaptation to the new metabolic needs [24].

Here we evaluated the impact of KSHV-mediated AKT hyperphosphorylation in THP-1 infected cells and how it could be possible to inhibit this pathway. We show that KSHV-latent infection of THP-1 cells resulted in AKT hyperactivation that correlated with an higher resistance to the treatment with proteasome inhibitor bortezomib, whose cytotoxic effect can be mediated also by reducing AKT phosphorylation in several tumor cell types [25-27]. AKT hyperphosphorylation by KSHV correlated with GLUT1 plasma-membrane exposure on the cell surface in THP-1 cells. Treatment of THP-1 infected cells or Primary Effusion Lymphoma (PEL) cells, harboring KSHV, with 2-Deoxy-D-glucose (2DG), a glycolysis inhibitor reported to induce a cytotoxic effect in cancer cells [28], allowed efficient cell death that was further increased by combination with bortezomib. Our study reinforces the growing interest of metabolic perturbation in cancer therapy and highlights the potential use of the combination of bortezomib and 2DG as an anticancer treatment of KSHV-associated malignancies.

## Materials and methods

### Cell cultures and reagents

Human monocytic cell line THP-1 and primary effusion lymphoma (PEL) were cultured in RPMI 1640 (Sigma, St. Louis, MO, USA; cat no. R0883) supplemented with 10% fetal bovine serum (Euroclone, Milan, Italy; cat no. ECLS0180L), glutamine (300 g/ml), streptomycin (100 g/ml) and penicillin (100U/ml, Gibco Carlsbad, CA, USA; cat no. 10378-016) in 5% CO_2_ at 37°C.

2-Deoxy-D-glucose (2DG) (Sigma cat no. D8375) was used at 10mM, Bortezomib (Santa Cruz, CA, USA; cat no. sc-217785) and AKT inhibitor LY294002 (Sigma cat no. P0037) were used at concentration of 10 nM and 1 μM respectively.

### Virus and infection

KSHV virus produced from BCBL-1 cell line was used to infect THP-1 cells, as previously reported [29]. Briefly, THP-1 cells were pelleted and incubated with KSHV (200X) at 37°C for 1h. Cells were then plated in complete medium and used for further treatments.

### Cell viability analysis

Cells were seeded in 24-well plates in complete medium and treated with Ly294002 (10μM), bortezomib (10nM), 2DG (10 mM) or 2DG (10 mM)/bortezomib (10nM). When LY294002 and bortezomib were used in combination, cells were pretreated with LY294002 for 40 min before adding bortezomib. After 24h or 48h of treatment (for BCBL-1 and THP1 respectively) cells were collected, counted by trypan-blue exclusion assay using a hemocytometer; cell pellets were used for western blot analysis. Each experiment was performed in triplicate.

### Western blot analysis

Western Blot analysis was performed as described elsewhere [30]. Briefly, cell were lysed in modified RIPA buffer (150 mM NaCl, 1% NP40, 50 mM Tris–HCl pH8, 0,5% deoxycholic acid, 0,1% SDS, 1% Triton X-100 protease and phosphatase inhibitor), equal amount of lysates were loaded on 4-12% NuPage Bis tris gels (Life technologies cat no. NO0322BOX) electrophoresed and transferred to Nitrocellulose membrane (Whatman, GE Healthcare, cat. no. 10401196). Membranes were then blocked for 30 min at RT in PBS containing BSA 3% and 0,2% Tween-20 and then probed with primary antibody overnight at 4°C. After 3 washes in PBS-0,2% Tween 20, membranes were incubated for 45 min with the appropriate horseradish peroxidase-conjugated secondary antibody (Santa Cruz biotechnologies) then washed as described before and the blots were developed using ECL Blotting Substrate (Thermo Scientific, Rockford, IL, USA; cat no. 32209).

The following antibodies were used: mouse monoclonal anti β-actin (Sigma cat. no. A2228), rabbit polyclonal anti Phospho-Akt (Ser473) (Cell Signaling cat.9271), rabbit polyclonal anti Akt (Cell Signaling cat.9272), rabbit polyclonal anti cleaved PARP (p-85, cell signaling cat. 9542), rabbit polyclonal anti GLUT1 (Santa Cruz cat no. sc-7903).

### Immunofluorescence

Cells were seeded on multispot slides, fixed for 10 min in cold methanol (−20°C) and incubated with the following primary antibodies for 1h at room temperature (RT): mouse anti LANA (Novus Biologicals cat no. NBP1-30176) and rabbit anti GLUT-1 (Santa Cruz cat no. sc-7903). After incubation with appropriate conjugate secondary antibody (30 min at RT), cell were stained with DAPI. Finally, microscope slides were mounted using PBS- Glicerol 1:1 and visualized by a Apotome Axio Observer Z1 inverted microscope (Zeiss, Oberkochen, Germany), equipped with an AxioCam MRM Rev.3 camera at 40 × magnification.

### Cell fractionation and membrane preparation

Cell fractionation was performed as described elsewhere [31]. Briefly, treated and untreated THP-1 cells were harvested, washed with PBS and resuspended in HEM buffer (20 mM HEPES (*N*-2-hydroxyethylpiperazine-*N*’-2-ethanesulfonic acid), 1 mM EDTA, 1 mM 2-mercaptoethanol and protease inhibitors). Cells were Dounce homogenized and nuclei were collected by centrifugation at 750 × *g* for 5 min. Cell extracts were kept at 4°C for 5 min and the remaining intact nuclei were collected by a further centrifugation at 750 × *g* for 5 min. The supernatant was recovered and a crude membrane fraction was obtained by centrifugation at 43,000 × *g* for 20 min. The leftover supernatant represented the cytoplasmic fraction. Nuclear and membrane fractions were than separated on SDS-PAGE, transferred to nitrocellulose membrane (GE Healthcare) and analyzed by western blot with the appropriate antibodies.

### Statistics

All experiment unless indicated were performed at least three times. All experimental results were expressed as the arithmetic mean ± standard deviation (s.d.). Student’s *t*-test was used for statistical significance of the differences between treatment groups. Statistical analysis was performed using analysis of variance at 5% (p < 0.05) or 1% (p < 0.01).

## Results and discussion

### KSHV-latent infection of monocytic cell line THP-1 results in an increase of AKT phosphorylation that persisted after bortezomib treatment

**Figure 1:**
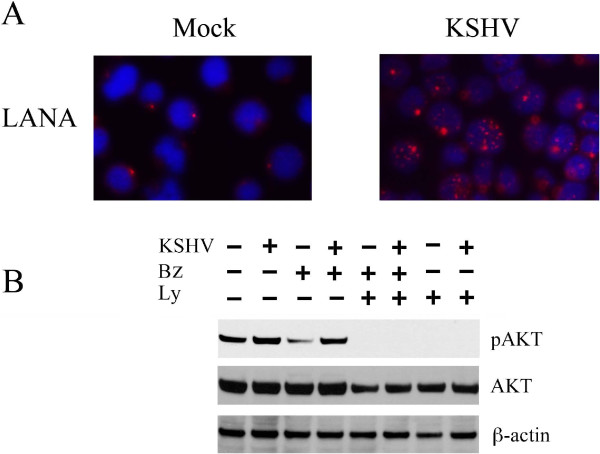
**Hyperphosphorylation of Akt induced by KSHV in THP-1 infected cells is resistant to Bortezomib treatment. A)** Immunofluorescence of mock and KSHV-infected THP-1 cells with anti-LANA antibodies. Typical LANA staining (intranuclear red punctuation) is visible in cells latently infected by KSHV. The counterstaining of THP-1 DNA with DAPI (blue) is shown. **B)** Western blot analysis of phospho-Akt (p-AKT) and total AKT (AKT) in mock and KSHV-infected THP-1 cells, untreated or treated with Bortezomib (Bz, 10 nM), or LY294002 (Ly, 1μM) or combination of both (Bz, 10 nM plus Ly, 1μM). β-actin is included as protein loading control.

THP-1 monocytic cells, infected with KHSV for 48 hours, were subjected to immunofluorescence analysis and, as shown in Figure 1A, the expression of latent associated nuclear antigen (LANA) was detected in about 35% of the cells, compared to mock infected cells. No expression of lytic antigens was found (data not shown), in accordance to previous reported studies [12], indicating that KSHV establishes a latent infection in THP-1 cells. Next, we investigated the impact of KHSV-infection on AKT phosphorylation in THP-1 cells. Western blot analysis showed that THP-1-infected cells displayed increased phosphorylation of AKT, in comparison to THP-1 mock-infected cells (Figure 1B). This is in agreement with other studies showing that KSHV proteins are able to activate PI3K/AKT pathway or down-regulate AKT phosphatases such as PTEN in several cell types [14,20]. The activation of AKT pathway has been also reported for other oncoviruses [32]. As bortezomib has been shown to interfere with the activation status of AKT [27,33], we then investigated if bortezomib-treatment could affect AKT phosphorylation in THP-1 cells. We observed that bortezomib (Bz, 10 nM for 48 hours) strongly down-regulated AKT phosphorylation in mock-infected cells, while KSHV infection impaired such effect (Figure 1B). This might be due to KSHV-induced inhibition of PTEN, demonstrated in other studies [20], that could counteract the bortezomib-mediated up-regulation of this phosphatase [34]. As expected, AKT phosporylation was completely abolished by pre-treatment with AKT inhibitor LY294002, both in mock and viral-infected cells (Figure 1B). By inhibiting AKT phosphorylation we also observed a reduction of the total AKT protein, likely due to its reduced stability in the unphosphorylated state. Similar results were obtained inhibiting AKT phosphorylation with mTOR kinase inhibitor PP242 (data not shown).

### KSHV-mediated AKT hyperphosphorylation correlates with a reduction of bortezomib cytotoxic effect

**Figure 2:**
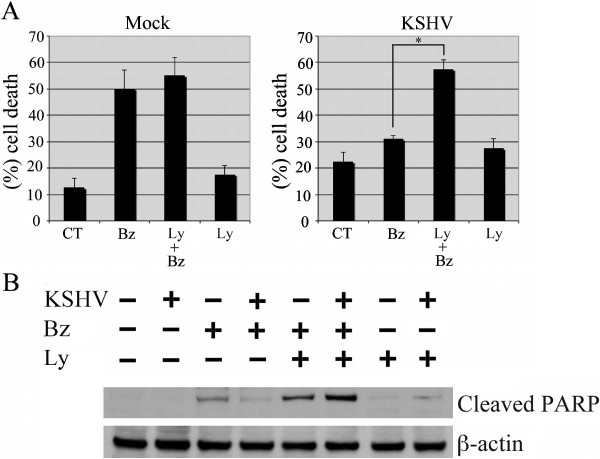
**KSHV-mediated AKT hyperphosphorylation correlates with a reduction of Bortezomib cytotoxic effect. A)** THP-1 mock and KSHV-infected cells were treated with bortezomib (Bz,10nM, for 48h) or AKT inhibitor LY294002 (Ly, 1μM) or combination of both (Bz, 10 nM plus Ly, 1μM). Cell death measurements were assayed by trypan-blue staining. The result is the mean ± SD of three independent experiments performed in duplicates. *p = 0.01. **B)** Western blot analysis of Cleaved-PARP of mock and THP-1-KSHV-infected cells untreated or treated with bortezomib (BZ), LY294002 (Ly) or both (Ly + BZ). β-actin is included as protein loading control.

One of the main molecular events of the bortezomib-induced cytotoxic effect is the down-regulation of AKT-phosphorylation, that can also be considered a biomarker for predicting chemoterapeutic response in some tumors [27,33]. Hence, we next investigated the biological effect of bortezomib-treatment with or without AKT inhibitor LY294002. The results, obtained by a trypan-blue exclusion viability assay, indicated that 10 nM bortezomib efficiently induced THP-1 mock-infected cell death that was not further increased by combination with AKT inhibitor LY294002 (Figure 2A). In contrast, the negligible cell death induced by bortezomib in THP-1 KSHV-infected cells was significantly increased by AKT inhibitor LY294002 (Figure 2A). These data are in accordance with modification of AKT phosphorylation seen in Figure 1B. Moreover, apoptotic marker PARP cleavage was induced in bortezomib-treated mock-infected THP-1 cells and slightly increased by combination with AKT inhibitor LY294002 (Figure 2B). On the contrary, the impairment of PARP cleavage upon bortezomib treatment in KSHV-infected cells was efficiently reverted by combination with LY294002 (Figure 2B), confirming the role of AKT activation in the resistance to bortezomib treatment of THP-1 KSHV-infected cells. These results suggest the possibility to increase the bortezomib-cytotoxic effect by counteracting the KSHV-mediated AKT hyperactivation in THP-1 monocytic cells. The importance of the activation of AKT pathway in the control of cell survival has been previously reported in other lymphoma cell lines [35].

### AKT hyperactivation by KSHV is responsible for GLUT 1 membrane exposure, particularly during bortezomib-treatment

**Figure 3:**
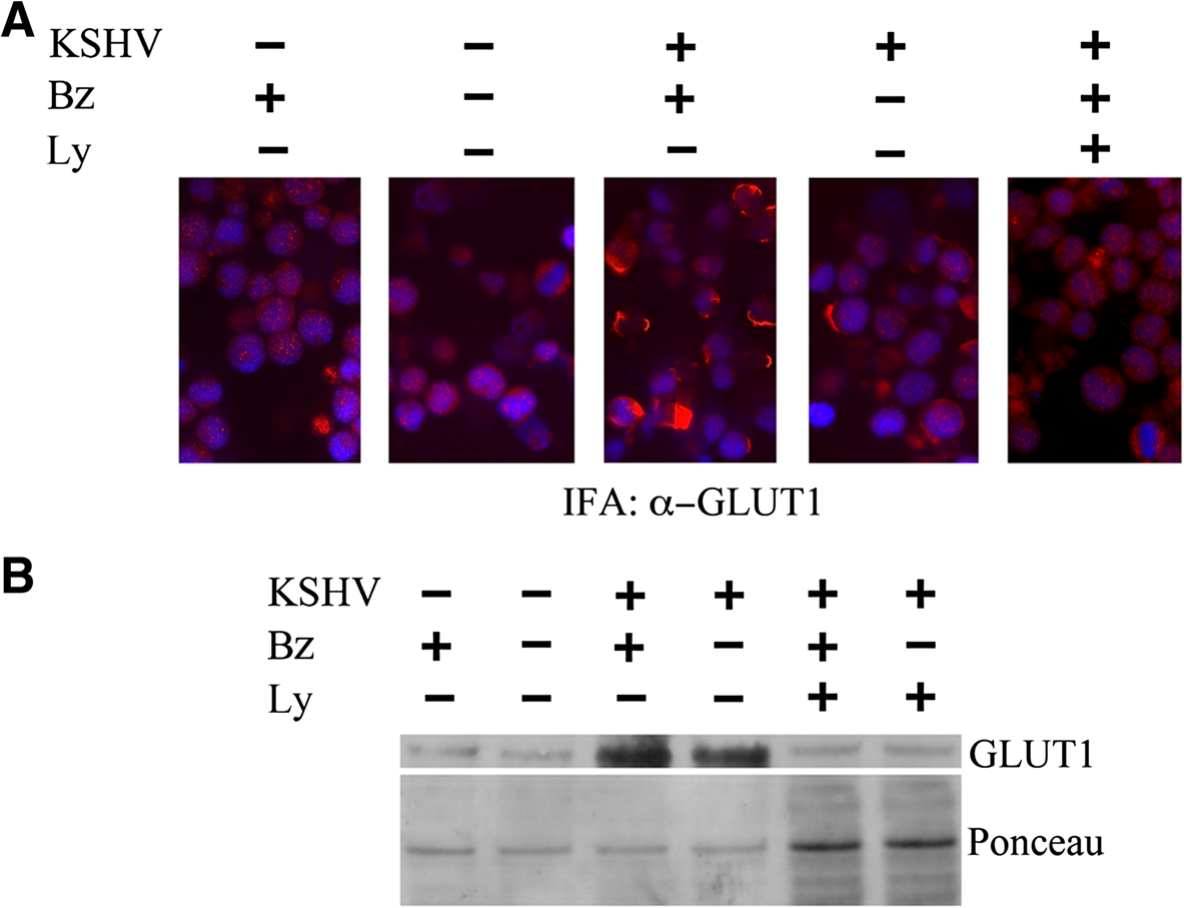
**GLUT1 membrane exposure, induced by KSHV infection of THP-1 cells, increases after Bortezomib treatment. A)** GLUT1 Immunofluorescence in mock and KSHV-infected THP-1 cells in the presence of Bortezomib (Bz), LY294002 (Ly) or the combination of them (Ly + Bz). GLUT1 staining (red) is mainly accumulated at the membranes on ~ 15% of KSHV-infected cells mock treated and in ~ 40% of the KSHV-infected cells upon bortezomib treatment. The counterstaining of THP-1 DNA with DAPI (blue) is shown. **B)** Western blot analysis showing the expression of GLUT1 in membrane fraction of mock and KSHV-infected THP-1 cells untreated or treated with bortezomib (Bz), LY294002 (Ly) or both (Ly + Bz). Ponceau staining of the membrane is reported as loading control.

The activation of PI3K/AKT pathway in cancer cells has been shown to influence the plasma membrane trafficking of one of the most ubiquitous glucose transporter molecule such as GLUT1 [36,37]. The exposure of GLUT1 on the cell surface up-regulates the glucose influx into the cells and gives a proliferating advantage to cells such as cancer cells that use this molecule as principal energetic source. This effect, described long time ago as Warburg effect [38], indicates the dependance of cancer cells on glycolysis also in aerobic conditions and helps these cells to survive in the hypoxic conditions typical of tumor microenviroment. KSHV has been previously reported to induce Warburg effect in endothelial cells through AKT activation and also a metabolic reprogramming in PEL cells [39,40]. An alteration of glucose metabolism has been described also for other oncogenic viruses [41,42]. Immunofluorescence analysis shows that KSHV infection (KSHV+) induced GLUT1 exposure on THP-1 cell membranes, compared to mock-infected cells (KSHV -), that was further increased following bortezomib treatment (Figure 3A). In agreement with the virus-induced AKT phosphorylation, GLUT1 membrane exposure was blocked by bortezomib combination with AKT inhibitor LY294002 in KSHV-infected THP-1 cells (Figure 3A).

Finally, the increase of GLUT1 membrane expression induced by KSHV in THP-1 was confirmed by western blot analysis of membrane extracts of infected and uninfected cells (Figure 3B). According to the immunofluorescence results, bortezomib treatment further increased the membrane expression of GLUT1 in THP-1-KSHV-infected cells, likely due to the inhibition of its proteasomal degradation mediated by bortezomib. GLUT1 exposure was completely abolished by pre-treatment with AKT inhibitor LY294002 (Figure 3B). As equal loading control, the ponceau membrane staining was included.

### KSHV-infection induces 2-Deoxy-D-glucose cytoxicity, further increased by its combination with bortezomib

**Figure 4:**
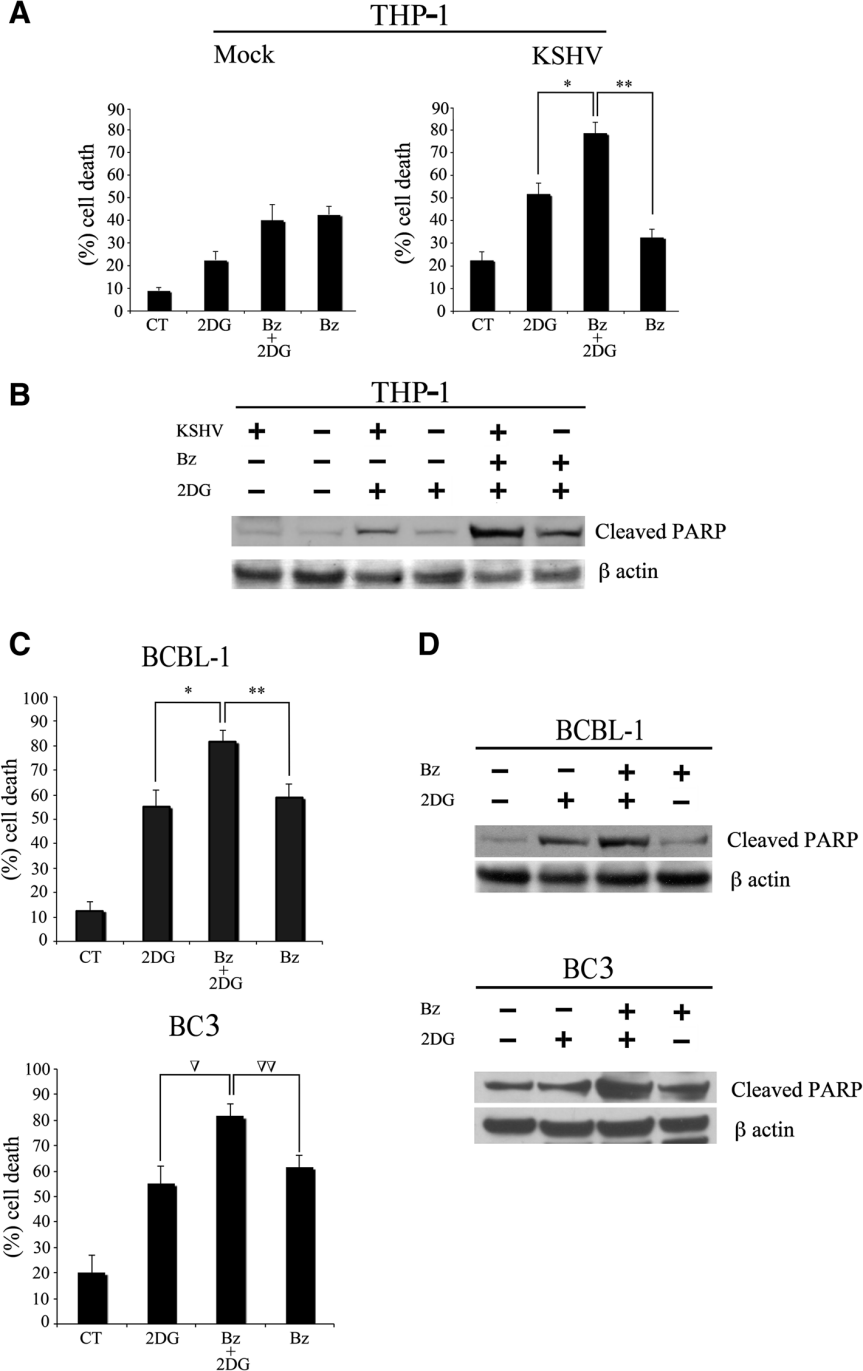
**KSHV latent infection induces 2-Deoxy-D-glucose cytotoxicity, further increased by its combination with bortezomib. A)** THP-1 mock and KSHV-infected cells were treated with bortezomib (BZ, 10 nM, for 48h) with or without glycolysis inhibitor 2DG (10 mM). Cell death measurements were assayed by trypan-blue staining. The result is the mean ± SD of three independent experiments performed in duplicates. *p = 0.01; **p = 0.001. **B)** Western blot analysis showing the expression of cleaved PARP in THP-1 mock and KSHV-infected cells treated with 2DG, Bz and 2DG + Bz. β-actin is included as protein loading control. **C)** BCBL1 and BC3 PEL cells were treated with bortezomib (Bz, 10 nM, for 48h) with or without glycolysis inhibitor 2DG (10 mM). Cell death measurements were assayed by tripan blue staining. The result is the mean ± SD of three indipendent experiments performed in duplicates. *p = 0.01, **p = 0.001; ∇p < 0.05, ∇∇p =0.05. **D)** Western blot analysis showing the expression of cleaved PARP in BCBL-1 and BC3 cells following treatment with 2DG, 2DG + Bz and Bz. β-actin is included as protein loading control.

Cancer cells displaying elevated membrane expression of GLUT1 are highly dependent on glycolysis for their survival, therefore, glycolysis inhibition is an interesting anticancer strategy [23]. To test this outcome, we exposed THP-1 KSHV-infected cells to the glycolysis inhibitor 2-Deoxy-D-glucose **(**2DG) with or without bortezomib treatment. We found that blocking glycolysis with 2DG treatment induced cell death in THP-1 infected cells and to a lesser extent also in the mock infected cells (Figure 4A). Interestingly though, 2DG treatment significantly increased bortezomib-induced cell death in KSHV-infected THP-1 cells, while it did not further increase the bortezomib-induced cell death in mock-infected cells (Figure 4A). Similar results were also obtained in BCBL-1 and BC3 primary effusion lymphoma (PEL) cell lines, that are latently infected by KSHV (Figure 4C). We previously reported that bortezomib induced immunogenic cell death in BCBL-1 cells [43,44] and here we found that such a cell death was significantly increased following 2DG co-treatment that was also cytotoxic by itself (Figure 4C). The cell death results, in THP-1, BCBL-1 and BC3 cells were confirmed by western immunoblotting of PARP cleavage, as shown in Figure 4B and D. These findings strengthen the use of glycolysis inhibition in combination with Bz in the KSHV de novo infected cells and in KSHV-associated tumor cells.

## Conclusions

The knowledge of the pathways and their downstream effectors that confer a growth advantage to cancer cells is of pivotal importance in the attempt to revert their pro-survival effects into an Achilles’ heel. Our results indicate that KSHV increases the oncogenic potential of the THP1-infected cells by hyper-activating PI3K/AKT pathway. This leads to an increase of bortezomib-resistance and to a GLUT1 plasma-membrane exposure. However we found that these pro-survival effects turned out to be detrimental for cell survival when AKT or glycolysis inhibitors were used, particularly in combination with bortezomib. These data encourage the use of such a combination treatment as a therapeutic strategy against KSHV associated malignancies.

## Abbreviations

2DG: 2-Deoxy-D-glucose; GLUT1: Glucose transporter 1; KSHV: Kaposi sarcoma-associated herpesvirus; LANA: Latency-associated nuclear antigen; MCD: Multicentric Castleman’s disease; PEL: Primary effusion lymphoma; PI3K: Phosphatidylinositol-3-kinase; PIP2: Phosphatidylinositol-4,5-bisphosphate; PIP3: Phosphatidylinositol-3,4,5-triphosphate; PTEN: Phosphatase and tensin homolog; Bz: Bortezomib.

## Competing interests

The authors declare that they have no competing interests.

## Authors’ contributions

Conceived the experiments: MC, RG, RS. Performed Western blot analysis: RG, AF and MG. Performed Immunofluorescence analysis: RS, RG. Interpretation of results and wrote the paper: MC, AF, GDO. All authors read and approved the final manuscript.
